# Supplementary material for: Assessment of Hydrocarbon Degradation Potential in Microbial Communities in Arctic Sea Ice
Source: Microorganisms. 2022 Feb 1;10(2):328. doi: 10.3390/microorganisms10020328 (PMC8879337; doi:10.3390/microorganisms10020328)
Supplement: Supplementary file 1 [file microorganisms-10-00328-s001.zip › microorganisms-1547119-supplementary.pdf]

Genome-resolved Metagenomics of Oil Degrading Microbial Community in Arctic Seaice

Supplementary tables:
Table S1. The characteristics of metagenomic data of seawater (SW), sea ice (SI), and crude oil encapsulating sea ice (SIO).
Table S2. List of bacterial genera containing oil hydrocarbon degrading organisms added to the HDO database from this study.
Table S3. List of targeted hydrocarbon degradation genes.
Table S4. The proportions of taxonomically assigned sequences at kingdom, phylum (>1%), and class (>1%) level from all sequences, and on genus level from prokaryotic sequences in SW, SI, and SIO metagenomes.
Table S5. The proportions of 20 most abundant prokaryotic genera associated with degradation of oil hydrocarbons from prokaryotic sequences in SW, SI, and SIO metagenomes.
Table S6. Normalized counts of genes associeted with degradation of alkanes in prokaryotic community of SW, SI, and SIO.
Table S7. Normalized counts of genes associated with monocyclic aromatic hydrocarbons degradation in prokaryotic community of SW, SI, and SIO.
Table S8. Normalized counts of genes associated with polycyclic aromatic hydrocarbons degradation in prokaryotic community of SW, SI, and SIO.
Table S9. Normalized counts of genes associated with various types of hydrocarbons degradation in prokaryotic community of SW, SI, and SIO.
Table S10. List of prokaryotic genera associated with one or more genes from the group of alkane degradation genes according to the affiliation of contigs of SW, SI, and SIO metagenomes.
Table S11. List of top100 prokaryotic genera associated with one or more genes from the group of monocyclic aromatic hydrocarbons degradation genes accordding to the affiliation of contigs of SW, SI, and SIO metagenomes.
Table S12. List of prokaryotic genera associated with one or more genes from the group of polycyclic aromatic hydrocarbons degradation genes according to the affiliation of the contigs of SW, SI, and SIO metagenomes.
Table S13. List of top100 prokaryotic genera associated with one or more genes from the group of genes related to the degradatio of various types of hydrocarbons according to the affiliation of contigs of SW, SI, and SIO metagenomes.
Table S14. The characteristics of all metagenome assembled genomes (MAGs) recovered from SW, SI, and SIO metagenomes.
Table S15. Taxonomic classification of good quality MAGs assembled from metagenomes of SW, SI, and SIO.

Supplementary figures:
Figure S1. The proportions of archaeal phyla, and the dominant archaeal genera in the prokaryotic community of SW, SI, and SIO.

Table S1. The numbers of total reads, and numbers and proportions (in parenthesis) of reads after the quality filtering for metagenomes obtained from the samples collected at different times (1 in superscript marks October and 2 in superscript November 2015), as well as the numbers of classified reads, metrics for coverage and diversity, and numbers of bacterial (b) archaeal (a) and unclassified (uc) contigs in pooled metagenomes of seawater (SW), sea ice (SI) and crude oil encapsulating sea ice (SIO).

**Sample code**	**Number of reads** (R1.fq +R2.fq)	**Number of reads after quality filtering**	**Number of classified reads**	**Coverage**	**Diversity**	**Number of contigs**
**SW**[1]	4788884 (1.3G + 1.4G)	4270304 (90.8%)	3892126	0.58	20.41	**75931** b_62996 a_2282 uc_4155
**SW**[2]	2509372 (2.1G + 2.2G)	2389475 (95.2%)				
**SI**[1]	2979989 (1.4G + 1.5G)	2799244 (93.9%)	4108966	0.58	20.40	**84483** b_74947 a_1297 uc_3316
**SI**[2]	3128415 (1.4G + 1.7G)	2947113 (94.2%)				
**SIO**[1]	2174740 (0.917G + 0.949G)	1946867 (89.5%)	2726111	0.56	20.09	**51480** b_40339 a_1191 uc_3275
**SIO**[2]	2250415 (1.1G + 1.2G)	2042199 (90.8%)				

Table S2. Bacterial genera added to the list of genera containing oil hydrocarbon degrading organisms proposed by Nõlvak et al., 2021.

**Genus**	**Class**	**Phylum**	**Reference**
Bermanella	Gammaproteobacteria	Proteobacteria	Hu et al., 2017
Dokdonia	Flavobacteriia	Bacteroidetes	Nõlvak et al., 2021
Gemmatimonas	Gemmatimonadetes	Gemmatimonadetes	Sampaio et al., 2017
Nonlabens	Flavobacteriia	Bacteroidetes	Kwon et al., 2013
Planktomarina	Alphaproteobacteria	Proteobacteria	Voget et al., 2015

Table S3. List of targeted hydrocarbon degradation genes with the respective encoded enzymes. In the case of abbreviations unique to this study, KEGG codes are provided in parentheses.

ALKANES DEGRADATION GENES

alkB1_2/*alkM*	alkane 1-monooxygenase
almA	flavin-binding monooxygenase
bmoB	butane monooxygenase regulatory protein
bmoC	butane monooxygenase regulatory protein
bmoD	butane monooxygenase hypothetical assembly protein (chaperonin-like protein BmoG)
bmoX	butane monooxygenase α subunit
bmoY	butane monooxygenase β subunit
bmoZ	butane monooxygenase γ subunit
ladA	long-chain alkane monooxygenase
MAH1	long-chain alkane monooxygenase
prmA	propane 2-monooxygenase large subunit
prmB	propane 2-monooxygenase reductase component
prmC	propane 2-monooxygenase small subunit
prmD	propane 2-monooxygenase coupling protein
rubB/*alkT*	rubredoxin---NAD+ reductase

MONOCYCLYC AROMATIC COMPOUNDS DEGRADATION GENES

a-adh (K00055)	aryl-alcohol dehydrogenase
**ADH1**	alcohol dehydrogenase class-P
bsdC2	4-hydroxybenzoate decarboxylase/phenol carboxylase subunit C
bsdC1	vanillate/4-hydroxybenzoate decarboxylase subunit C
bsdD	vanillate/4-hydroxybenzoate decarboxylase subunit D/p-hydroxybezoate decarboxylase
cymAa	p-cymene methyl-monooxygenase
cymAb	p-cymene methyl-monooxygenase electron transporter component
dmpK	phenol/toluene 2-monooxygenase (NADH) P0/A0 assembly subunit
dmpL	phenol/toluene 2-monooxygenase (NADH) P1/A1
dmpM	phenol/toluene 2-monooxygenase (NADH) P2/A2
dmpN	phenol/toluene 2-monooxygenase (NADH) P3/A3
dmpO	phenol/toluene 2-monooxygenase (NADH) P4/A4
dmpP	phenol/toluene 2-monooxygenase (NADH) P5/A5 ferredoxin subunit
etbAa	ethylbenzene dioxygenase subunit α
etbAb	ethylbenzene dioxygenase subunit β
etbAc	ethylbenzene dioxygenase ferrodoxin component
hcaB	2,3-dihydroxy-2,3-dihydrophenylpropionate dihydrogenase
hcaC	3-phenylpropionate/trans-cinnamate dioxygenase ferredoxin component
hcaD	3-phenylpropionate/trans-cinnamate dioxygenase ferredoxin reductase component
hcaE	3-phenylpropionate/trans-cinnamate dioxygenase subunit α
hcaF	3-phenylpropionate/trans-cinnamate dioxygenase subunit β
pchC	4-cresol dehydrogenase (hydroxylating) cytochrome subunit

pchF	4-cresol dehydrogenase (hydroxylating) flavoprotein subunit
ped	(s)-1-phenylethanol dehydrogenase
phe (K03380)	phenol 2-monooxygenase (NADPH)
tmoA	toluene monooxygenase system protein A
tmoB	toluene monooxygenase system protein B
tmoC	toluene monooxygenase system ferredoxin subunit
tmoD	toluene monooxygenase system protein D
tmoE	toluene monooxygenase system protein E
tmoF	toluene monooxygenase electron transfer component
todA	benzene/toluene/chlorobenzene dioxygenase ferredoxin reductase component
todB	benzene/toluene/chlorobenzene dioxygenase ferredoxin component
todC1	benzene/toluene/chlorobenzene dioxygenase subunit α
todC2	benzene/toluene/chlorobenzene dioxygenase subunit β
xylA	toluene methyl-monooxygenase electron transporter complex
xylM	toluene methyl-monooxygenase
xylC	benzaldehyde dehydrogenase (NAD)

POLYCYCLYC AROMATIC COMPOUNDS DEGRADATION GENES

CYP1A1	cytochrome P450 fam. 1 subfam. A1
CYP1A2	cytochrome P450 fam. 1 subfam. A2
CYP2A6	cytochrome P450 fam. 2 subfamily A6
CYP3A4	cytochrome P450 fam. 3 subfam. A4
gst	glutation s-transferase
GSTK1	glutation s-transferase kappa 1
HPGDS	prostaglandin-H2 D-isomerase
nahB	cis-1,2-dihydroxynaphthalene/dibenzothiophene dihydrodiol dehydrogenase
nahC	1,2-dihydroxynaphthalene dioxygenase
nidA	PAH dehydrogenase large subunit
nidB	PAH dehydrogenase small subunit
phdK	2-formylbenzoate dehydrogenase

VARIOUS COMPOUNDS DEGRADATION GENES

adh	alcohol dehydrogenase
adhE	acetaldehyde dehydrogenase / alcohol dehydrogenase
adhP	alcohol dehydrogenase propanol-preferring
**ALDH**	aldehyde dehydrogenase (NAD+)
chnB	cyclohexanone monooxygenase
dbfA1	dibenzofuran dioxygenase subunit α
dbfA2	dibenzofuran dioxygenase subunit β
frmA	s-(hydroxymethyl)glutathione dehydrogenase /alcohol dehydrogenase
ligA	protocatechuate 4,5-dioxygenas subunit α
ligB	protocatechuate 4,5-dioxygenas β chain
nagG	salicylate 5-hydroxylase large subunit
nagH	salicylate 5-hydroxylase small subunit

nahAa	naphthalene 1,2-dioxygenase ferredoxin reductase component
nahAb	naphthalene 1,2-dioxygenase ferredoxin component
nahAc	naphthalene 1,2-dioxygenase subunit α
nahAd	naphthalene 1,2-dioxygenase subunit β
nmsA	naphtyl-2-methyl-succinate synthase α subunit
nmsB	naphtyl-2-methyl-succinate synthase β subunit
nmsC	naphtyl-2-methyl-succinate synthase γ subunit
pcaG	protocatechuate 3,4-dioxygenas α subunit
pcaH	protocatechuate 3,4-dioxygenas β subunit
phdI	1-hydroxy-2-naphthoate dioxygenase
phdJ	4-(2-carboxyphenyl)-2-oxobut-3-enoate aldolase
phdE	cis-3,4-dihydrophenanthrene-3,4-diol dehydrogenase
salDH (K00480)	salicylate dehydrogenase
yiaY	alcohol dehydrogenase

ANAEROBIC OIL COMPOUNDS DEGRADATION GENES

benS (K07540)	benzylsuccinate synthase

Table S4. The proportions (%) of taxonomically assigned sequences at kingdom, phylum (>1%), and class (>1%) level from all sequences of seawater (SW), sea ice (SI), and crude oil encapsulating sea ice (SIO) metagenomes. The proportions (%) at genus (20 most abundant in each sample) level are calculated from all prokaryotic sequences and marked with grey background. Microbial genera shown in bold are genera known to contain hydrocarbon degrading organisms according to the literature. co – cut off value.

Taxonomic level				SW (%)	SI (%)	SIO (%)
**Kingdom**	**Phylum**	**Class**	**Genus**			
Bacteria				66.21	78.51	74.22
	Actinobacteria			8.50	3.35	5.67
			Ca. Actinomarina	0.76	0.19	0.51
			Ca. Aquiluna	0.11	< co	< co
			Streptomyces	0.16	< co	< co
	Bacteroidetes			10.24	5.39	4.86
			Flavobacterium	0.18	< co	< co
			Formosa	0.15	< co	< co
			Polaribacter	0.11	< co	< co
			Ulvibacter	0.10	< co	< co
	Cyanobacteria			1.96	< co	< co
			Synechococcus	1.67	0.53	0.30
	Planctomycetes			2.61	1.67	1.10
	Proteobacteria			37.86	62.75	58.26
		Alphaproteobacteria		21.29	35.40	33.92
			Ca. Endolissoclinum	0.35	1.32	1.15
			Ca. Pelagibacter	4.80	10.91	9.72
			Ca. Puniceispirillum	0.39	0.54	0.45
			Octadecabacter	0.13	< co	< co
			Ascidiaceihabitans	< co	0.13	0.37
			Planktomarina	1.58	1.52	1.12
			Roseovarius	0.11	0.17	0.21
			Ruegeria	< co	0.13	0.18
			Sulfitobacter	0.19	0.41	0.87

			Tateyamaria	< co	< co	0.17
		Betaproteobacteria		2.93	4.36	3.57
			Limnohabitans	0.09	< co	< co
			Polynucleobacter	0.16	0.21	< co
		Gammaproteobacteria		11.95	20.75	18.48
			Alteromonas	< co	0.18	0.18
			Bermanella	< co	0.35	0.84
			Ca. Thioglobus	1.06	2.60	1.71
			Colwellia	< co	0.37	1.74
			Glaciecola	< co	0.42	1.05
			Marinomonas	< co	0.18	< co
			Pseudoalteromonas	< co	0.16	0.28
			Pseudomonas	< co	0.26	0.29
			Vibrio	< co	0.14	0.19
		Other Proteobacteria		1.70	2.24	2.29
	Verrucomicrobia			1.68	1.88	1.24
	Other bacteria			3.35	3.47	3.10
Archaea				4.33	1.16	1.39
			Nitrosopumilus	0.09	< co	< co
Unclassified				29.46	20.32	24.39

Table S5. The proportions (%) of seguencing of 20 most abundant prokaryotic genera known to contain hydrocarbon degrading organisms from prokaryotic sequences in metagenomes of seawater (SW), sea ice (SI), and crude oil encapsulating sea ice (SIO). co – cut off value.

Genus	SW	SI	SIO
Alteromonas	< co	0.098	0.18
Bacillus	0.080	< co	0.091
Bermanella	< co	0.35	0.84
Bradyrhizobium	0.070	0.097	0.093
Colwellia	< co	0.37	1.74
Flavobacterium	0.18	0.098	< co
Glaciecola	< co	0.42	1.05
Legionella	0.071	< co	< co
Maribacter	0.068	< co	< co
Marinomonas	< co	0.18	0.11
Mesorhizobium	0.08	0.10	0.11
Oleispira	< co	0.10	0.10
Paracoccus	0.060	< co	< co
Paraglaciecola	< co	< co	0.097
Planktomarina	1.58	1.52	1.11
Polaribacter	0.11	< co	< co
Pseudoalteromonas	0.081	0.16	0.28
Pseudomonas	0.20	0.27	0.29
Rhizobium	0.058	< co	< co
Rhodobacter	0.072	0.090	< co
Roseobacter	< co	0.082	0.13
Roseovarius	0.11	0.17	0.21
Ruegeria	0.087	0.13	0.18
Shewanella	< co	< co	0.093
Sphingomonas	0.073	0.092	< co
Streptomyces	0.16	0.11	0.11
Sulfitobacter	0.19	0.41	0.87
Ulvibacter	0.10	< co	< co
Vibrio	0.10	0.14	0.19
**Total proportion**	**7.11**	**9.35**	**12.16**

Table S6. Normalized counts (RPKG) of genes associated with alkanes degradation in metagenomes of seawater (SW), sea ice (SI), and crude oil encapsulating sea ice (SIO). The names of respective encoded enzymes can be found in Table S3. Genes encoding multiple subunits of one enzyme are shaded with gray background. * The genes encoding subunits for electron transport and showing remarkably higher counts compared to the genes encoding other subunits of the same enzyme were not included in the analysis.

ALKANES DEGRADATION GENES															
	alkB1_2/ *alkM*	***almA***	***bmoB***	****bmoC***	***bmoD***	***bmoZ***	***bmoX***	***bmoY***	***ladA***	***MAH1***	***prmA***	****prmB***	***prmC***	***prmD***	***rubB/*** *alkT*
**SW**	0.39	1.35	0	0.49	0	0	0.01	0	0.56	0.21	0.01	2.51	0.02	0	3.55
**SI**	0.36	1.80	0	0.50	0	0	0.02	0	0.68	0.17	0.02	2.52	0.02	0	3.44
**SIO**	0.33	1.12	0	0.64	0	0	0	0	0.40	0.18	0.04	2.77	0	0	3.81
**Average counts of all genes encoding subunits of the enzymes**															
**SW**	-	-	0.002						-	-	0.01				-
**SI**	-	-	0.004						-	-	0.01				-
**SIO**	-	-	0						-	-	0.01				-
**Summarized counts of all genes**															
**SW**	6.07														
**SI**	6.46														
**SIO**	5.85														

Table S7. Normalized counts of genes (RPKG) related to the degradation of monocyclic aromatic hydrocarbons (MAHs) in metagenomes of seawater (SW), sea ice (SI), and sea ice encapsulating crude oil (SIO). The names of the respective enzymes or their subunits encoded by these genes can be found in Table S3. The genes encoding multiple subunits of the enzyme are shaded with gray background. * The genes encoding subunits for electron transport and showing remarkably higher counts compared to the genes encoding other subunits of the same enzyme were not included in the analysis.

MONOCYCLYC AROMATIC COMPOUNDS DEGRADATION GENES																																						
	a-adh (K00055)	**ADH1**	***bsdC***	***bsdC***	***bsdD***	***cymAa***	****cymAb***	***dmpK***	***dmpL***	***dmpM***	***dmpN***	***dmpO***	****dmpP***	***etbAa***	***etbAb***	***etbAc***	***hcaB***	****hcaC***	****hcaD***	***hcaE***	***hcaF***	***pchC***	***pchF***	***ped***	***phe*** (K03380)	***tmoA***	***tmoB***	****tmoC***	***tmoD***	***tmoE***	****tmoF***	***todA***	***todB***	***todC1***	***todC2***	**xylA**	***xylM***	***xylC***
**SW**	5.01	2.06	0.25	0.38	0	0.41	1.14	0.06	0.02	0.07	0.01	0	2.45	1.81	0.15	2.00	23.2	5.05	4.55	2.03	0.07	0	0.97	22.7	0.64	0.01	0.07	5.19	0.06	0.04	2.61	1.14	1.50	1.48	0.07	1.94	0.43	8.00
**SI**	6.08	2.67	0.42	0.74	0	0.30	1.18	0	0.02	0.06	0.02	0	2.33	2.05	0.28	1.86	26.8	5.66	4.02	2.23	0.25	0	1.33	26.0	0.94	0.02	0.11	5.92	0.05	0.03	2.57	1.28	1.30	1.74	0.17	1.94	0.33	7.91
**SIO**	5.09	2.06	0.20	0.22	0	0.28	1.38	0	0	0	0.04	0	2.57	1.16	0	0.97	21.4	3.63	4.34	1.32	0	0	0.95	20.4	0.71	0.04	0	3.74	0	0	2.83	1.48	0.72	0.81	0	2.23	0.31	8.03
**Average counts of genes encoding subunits of the enzymes**																																						
**SW**	-	-	-	0.19		0.41		0.03						1.32			-	1.05				0.49		-	-	0.05						1.05				1.19		-
**SI**	-	-	-	0.37		0.30		0.02						1.40			-	1.24				0.67		-	-	0.05						1.12				1.14		-
**SIO**	-	-	-	0.11		0.28		0.01						0.71			-	0.66				0.48		-	-	0.01						0.75				1.27		-
**Summarized counts of all genes**																																						
**SW**	44.44																																					
**SI**	50.33																																					
**SIO**	40.77																																					

Table S8. Normalized counts (RPKG) of genes related to the degradation of polycyclic aromatic hydrocarbons (PAHs) in metagenomes of seawater (SW), sea ice (SI), and crude oil encapsulating sea ice (SIO). The names of the respective enzymes or enzyme subunits encoded by these genes can be found in Table S3. The genes encoding subunits of one enzyme are shaded with gray background.

POLYCYCLYC AROMATIC COMPOUNDS DEGRADATION GENES (RPKG)												
	CYP1A1	***CYP1A2***	***CYP2A6***	***CYP3A4***	***gst***	***GSTK1***	***HPGDS***	***nahB***	***nahC***	***nidA***	***nidB***	***phdK***
**SW**	0.77	0.68	0.17	0.28	4.69	0.57	0.64	18.32	0.28	1.37	0.08	7.40
**SI**	0.80	0.58	0.13	0.20	7.29	0.82	1.15	21.01	0.35	1.61	0.19	7.16
**SIO**	0.46	0.40	0.14	0.15	5.36	0.69	0.99	16.48	0.19	0.72	0	7.16
**Average counts of genes encoding subunits of the enzymes**												
**SW**	0.73		-	-	-	-	-	-	-	0.73		-
**SI**	0.69		-	-	-	-	-	-	-	0.90		-
**SIO**	0.43		-	-	-	-	-	-	-	0.36		-
**Summarized counts of all genes**												
**SW**	33.81											
**SI**	39.70											
**SIO**	31.95											

Table S9. Normalized counts of genes (RPKG) associated with various types of hydrocarbons degradation in metagenomes of seawater (SW), sea ice (SI), and sea ice encapsulating crude oil (SIO) metagenomes. The names of the enzymes and enzyme subunits encoded by these genes can be found in Table S3. Genes encoding multiple subunits of the enzyme are shaded with gray background. * The genes encoding subunits for electron transport and showing remarkably higher counts compared to the genes encoding other subunits of the same enzyme were not included in the analysis.

VARIOUS COMPOUNDS DEGRADATION GENES (RPKG)																									
	adh	***adhE***	***adhP***	**ALDH**	***chnB***	***dbfA1***	***dbfA2***	***frmA***	***ligA***	***ligB***	***nagG***	***nagH***	****nahAa***	****nahAb***	***nahAc***	***nahAd***	***nmsA***	***nmsB***	***nmsC***	***pcaG***	***pcaH***	***phdI***	***phdJ***	***phdE***	***salDH*** (K00480)	***yiaY***
**SW**	6.67	2.30	5.46	4.86	1.47	1.65	0.15	2.62	0.19	0.10	1.88	0	2.64	3.23	1.47	0	0	0	0	0.61	0.28	0.18	0.93	10.7	2.41	1.07
**SI**	8.04	2.57	6.65	4.83	2.04	1.91	0.29	3.25	0.32	0.40	2.13	0.31	2.65	2.75	1.78	0.55	0	0	0	0.87	0.42	0.38	0.92	12.7	2.95	1.43
**SIO**	6.36	2.55	5.38	4.99	1.60	0.96	0	2.52	0.29	0.27	1.28	0.12	2.92	1.84	0.88	0	0	0	0	0.70	0.27	0.03	0.87	9.53	2.44	1.17
**Average counts of genes encoding subunits of the enzymes**																									
**SW**	-	-	-	-	-	0.9		-	0.15		0.94		0.36				0			0.45		0.56		-	-	-
**SI**	-	-	-	-	-	1.1		-	0.36		1.22		1.17				0			0.65		0.65		-	-	-
**SIO**	-	-	-	-	-	0.48		-	0.28		0.70		0.44				0			0.49		0.45		-	-	-
**Summarized counts all genes**																									
**SW**	40.92																								
**SI**	49.61																								
**SIO**	39.38																								

Table S10. Normalized counts (RPKG) of the sequences of prokaryotic genera associated with the genes or gene clusters from alkanes degradation gene group. The associations are based on the affiliation of contigs containing sequences of respective genes in seawater (SW), sea ice (SI), and crude oil encapsulating sea ice (SIO) metagenomes.

Phylum/class	Genus	Gene/gene cluster	Normalized counts		
			SW	SI	SIO
Alphaproteobacteria	*Octadecabacter*	*alkB1_2*	0.66	–	–
	Planktomarina		1.32	1.10	1
	Rhodobacter		0.66	–	–
	Sulfitobacter		–	0.55	–
	Tateyamaria		0.66	–	–
Gammaproteobacteria	*Bermanella*		–	1.10	3
	Endozoicomonas		–	–	1
	Methylophaga		0.66	–	–
	Oleispira		–	0.55	1
	Reinekea		–	0.55	–
Actinobacteria	*Acidimicrobium*		–	0.55	–
Bacteroidetes	*Altibacter*		0.66	–	–
	Kordia		–	0.55	–
	Winogradskyella		–	0.55	–
Alphaproteobacteria	*Bradyrhizobium*	*almA*	1.32	1.10	–
	Ca. Pelagibacter		4.64	2.76	2
	Sphingomonas		0.66	0.55	–
	Hyphomonas		0.66	0.55	–
	Ca. Puniceispirillum		0.66	–	–
	Thalassobaculum		0.66	–	–
	Reyranella		0.66	–	–
	Sulfitobacter		0.66	–	–
	Phaeobacter		–	0.55	–
	Pacificimonas		–	0.55	–
	Henriciella		–	0.55	–
	Planktotalea		–	–	1
Betaproteobacteria	*Ca. Methylopumilus*		0.66	–	–
Deltaproteobacteria	*Sandaracinus*		–	–	1
Gammaproteobacteria	*Marinobacter*		0.66	–	–
	Glaciecola		–	0.55	1
	Ca. Thioglobus		0.66	0.55	1
	Umboniibacter		0.66	0.55	–
	Shewanella		–	0.55	–
	Pseudomonas		–	0.55	–
	Bermanella		–	–	2
	Thalassotalea		–	–	1
Actinobacteria	*Ilumatobacter*		–	–	1
	Nocardioides		0.66	–	–
	Jatrophihabitans		0.66	–	–
	Nocardia		0.66	–	–
	Streptomonospora		0.66	0.55	–
	Streptomyces		0.66	–	–

Phylum/class	Genus	Gene/gene cluster	Normalized counts		
			SW	SI	SIO
Actinobacteria	*Saccharopolyspora*	*almA*	0.66	0.55	–
	Nonomuraea		0.66	0.55	–
	Cellulomonas		0.66	–	–
Cyanobacteria	*Trichodesmium*		–	0.55	–
Firmicutes	*Bacillus*		–	1.10	1
Gammaproteobacteria	*Ca. Thioglobus*	*bmoBCDXYZ*	0.66	1.10	–
Alphaproteobacteria	*Planktomarina*	*ladA*	0.66	0.55	1
	Roseicyclus		0.66	0.55	–
	Belnapia		0.66	–	–
	Planktotalea		0.66	–	–
	Reyranella		0.66	–	–
	Aureimonas		–	0.55	–
	Rhodoplanes		–	0.55	–
	Jannaschia		–	0.55	–
	Enhydrobacter		–	0.55	–
	Sulfitobacter		–	–	1
	Azospirillum		–	–	1
Gammaproteobacteria	*Microbulbifer*		–	0.55	–
Actinobacteria	*Mycolicibacterium*		–	0.55	–
Firmicutes	*Bacillus*		–	0.55	–
Alphaproteobacteria	*Ascidiaceihabitans*	*MAH1*	1.32	1.10	1
	Planktomarina		0.66	0.55	1
	Roseobacter		0.66	–	–
	Sulfitobacter		0.66	0.55	–
Cyanobacteria	*Bellilinea*		0.66	–	–
	Synechococcus		0.66	0.55	–
Firmicutes	*Paenibacillus*		0.66	0.55	–
Gammaproteobacteria	*Ca. Thioglobus*	*prmABCD*	1.32	1.10	1

Table S11. The normailized counts (RPKG) of the sequences of top100 prokaryotic genera associated with the genes and gene clusters from monocyclic aromatic hydrocarbons degradation gene group. The associations are based on the affiliation of the contigs containing sequences of respective genes in the seawater (SW), sea ice (SI), and crude oil encapsulating sea ice (SIO) metagenomes. Archaea are given on green background.

Phylum/class	Genus	Gene/gene cluster	Normalized counts		
			SW	SI	SIO
Gammaproteobacteria	*Bermanella*	*bsdC2*	–	1.10	1
	Colwellia		–	–	2
	Glaciecola		–	0.55	2
	Methylophaga		0.66	0.55	1
Cyanobacteria	*Synechococcus*		3.31	–	–
Thaumarchaeota	*Nitrosopumilus*		1.32	1.66	–
Gammaproteobacteria	*Methylophaga*	*bsdC1D*	0.66	0.55	1
	Bermanella		–	1.10	1
	Colwellia		–	0	2

Phylum/class	Genus	Gene/gene cluster	Normalized counts		
			SW	SI	SIO
	Glaciecola		–	0.55	2
Cyanobacteria	*Synechococcus*		3.97	–	–
Thaumarchaeota	*Ca. Nitrosomarinus*		0.66	–	1
	Nitrosopumilus		1.32	1.66	–
Alphaprotebacteria	*Planktomarina*	*cymAab*	0.66	0.55	1
Gammaproteobacteria	*Bermanella*		–	–	2
	Oleispira		–	0.55	1
Gammaproteobacteria	*Ca. Thioglobus*	*dmpKLMNOP*	0.66	1.10	1
Alphaprotebacteria	*Ca. Pelagibacter*	*etpAabc*	–	1.66	2
	Ca. Puniceispirillum		3.31	3.31	1
	Planktomarina		3.97	2.21	2
	Pseudooceanicola		0.66	0.55	1
	Pseudophaeobacter		–	0.55	1
	Roseovarius		0.66	1.10	–
Gammaproteobacteria	*Ca. Thioglobus*		7.28	9.39	4
Firmicutes	*Bacillus*		–	1.10	1
Alphaprotebacteria	*Ca. Pelagibacter*	*hcaCDEF*	5.30	4.42	7
	Ca. Puniceispirillum		3.31	3.31	1
	Planktomarina		3.97	2.21	2
	Pseudooceanicola		0.66	0.55	1
	Pseudophaeobacter		–	0.55	1
	Roseovarius		0.66	1.10	–
Gammaproteobacteria	*Ca. Thioglobus*		7.28	9.94	5
Cyanobacteria	*Synechococcus*		1.99	–	–
Firmicutes	*Bacillus*		–	1.10	1
Alphaprotebacteria	*Ca. Pelagibacter*	*pchCF*	3.31	2.76	1
	Emcibacter		0.66	0.55	1
	Planktomarina		3.97	3.31	2
	Roseobacter		0.66	–	1
	Salipiger		0.66	0.55	1
	Sulfitobacter		–	0.55	1
Betaproteobacteria	*Variovorax*		0.66	1.10	–
Gammaproteobacteria	*Bermanella*		–	0.55	1
	Ca. Thioglobus		1.32	6.63	10
	Glaciecola		–	0.55	1
	Pseudomonas		0.66	0.55	1
Cyanobacteria	*Synechococcus*		3.31	0.55	–
Gammaproteobacteria	*Ca. Thioglobus*	*tmoABCDEF*	1.32	1.10	1
Alphaprotebacteria	*Ca. Pelagibacter*	*todABC1C2*	4.64	3.31	7
	Ca. Puniceispirillum		2.65	1.66	1
	Planktomarina		3.97	2.21	2
	Pseudophaeobacter		–	0.55	1
	Roseovarius	*todABC1C2*	0.66	1.10	–
Gammaproteobacteria	*Ca. Thioglobus*		5.96	7.73	3
Firmicutes	*Bacillus*		–	1.10	1

Phylum/class	Genus	Gene/gene cluster	Normalized counts		
			SW	SI	SIO
Alphaprotebacteria	*Planktomarina*	*xylAM*	0.66	0.55	1
Gammaproteobacteria	*Bermanella*		–	0.55	2
	Oleispira		–	0.55	1
Alphaprotebacteria	*Antarctobacter*	*xylC*	–	0.55	1
	Ascidiaceihabitans		1.99	2.76	1
	Ca. Pelagibacter		40.40	46.41	65
	Ca. Puniceispirillum		3.97	4.42	1
	Cohaesibacter		0.66	–	1
	Fluviibacterium		–	0.55	1
	Kiloniella		0.66	1.10	–
	Leisingera		0.66	2.21	3
	Lentibacter		0.66	1.10	1
	Litoreibacter		0.66	2.21	–
	Maritalea		0.66	1.10	1
	Octadecabacter		1.32	1.10	–
	Pelagicola		–	0.55	1
	Planktomarina		18.54	14.92	25
	Pseudorhodobacter		1.32	0.55	–
	Rhodovulum		0.66	–	1
	Roseobacter		1.32	0.55	1
	Roseovarius		0.66	–	4
	Ruegeria		3.31	–	–
	Salinihabitans		0.66	–	1
	Shimia		1.32	0.55	–
	Sphingomonas		–	2.21	1
	Sulfitobacter		4.64	4.97	4
	Thalassobium		1.32	1.10	–
	Xanthobacter		0.66	0.55	1
	Yoonia		1.99	1.10	–
Betaproteobacteria	*Collimonas*		–	0.55	1
Epsilon	*Arcobacter*		–	–	2
Gammaproteobacteria	*Alteromonas*		–	0.55	1
	Amphritea		0.66	0.55	1
	Bermanella		–	2.21	8
	Ca. Thioglobus		13.91	20.44	17
	Colwellia		–	1.10	19
	Glaciecola		–	2.76	5
	Halioglobus		2.65	1.10	1
	Moritella		–	0.55	1
	Oceanospirillum		–	–	2
	Paraglaciecola		–	–	2
	Pseudoalteromonas		–	0.55	3
	Pseudomonas		1.99	1.66	1
	Rheinheimera		0	0.55	1
	Salinicola		0.66	1.66	–
Actinobacteria	*Ca. Aquiluna*		4.64	1.66	–

Phylum/class	Genus	Gene/gene cluster	Normalized counts		
			SW	SI	SIO
	Clavibacter		–	0.55	1
	Pontimonas		–	0.55	1
Bacteroidetes	*Chryseolinea*		0.66	1.10	–
	Polaribacter		0.66	0.55	1
Cyanobacteria	*Synechococcus*		4.64	0.55	–

Table S12. Normalized counts (RPKG) of sequences of prokaryotic genera associated with genes and gene clusters from polycyclic aromatic hydrocarbons degradation gene group. The associations are based on the affiliation of contigs containing sequences of respective genes in seawater (SW), sea ice (SI), and crude poil encapsulating sea ice (SIO) metagenomes.

Phylum/class	Genus	Gene	Normalized counts		
			SW	SI	SIO
Alphaproteobacteria	*Agrobacterium*	*CYP1A1A2*	–	0.55	–
	Aliiroseovarius		–	0.55	–
	Ascidiaceihabitans		–	1.66	1
	Labrenzia		–	0.55	–
	Novosphingobium		–	0.55	–
	Phenylobacterium		–	0.55	–
	Planktomarina		2.65	2.21	2
	Rhodobacter		–	0.55	1
	Roseobacter		0.66	–	–
	Sulfitobacter		–	0.55	–
	Tateyamaria		0.66	–	–
Betaproteobacteria	*Massilia*		–	0.55	–
Deltaproteobacteria	*Hyalangium*		0.66	–	–
Gammaproteobacteria	*Alteromonas*		–	0.55	–
	Halioglobus		1.32	0.55	–
	Marinobacter		–	–	1
	Marortus		–	0.55	–
	Photobacterium		–	–	1
	Pseudohaliea		0.66	–	–
Actinobacteria	*Ca. Actinomarina*		0.66	–	–
	Nakamurella		0.66	–	–
Chloroflexi	*Bellilinea*		0.66	–	–
Cyanobacteria	*Synechococcus*		3.31	1.10	–
Firmicutes	*Paenibacillus*		–	1.10	–
Verrucomicrobiae	*Verrucomicrobium*		0.66	–	–
Alphaproteobacteria	*Ascidiaceihabitans*	*CYP2A6*	–	0.55	1
	Planktomarina		0.66	0.55	1
	Roseobacter		0.66	–	–
Chloroflexi	*Bellilinea*		0.66	–	–
Firmicutes	*Paenibacillus*		–	0.55	–
Alphaproteobacteria	*Ascidiaceihabitans*	*CYP3A4*	–	1.10	1
	Planktomarina		0.66	0.55	1
	Roseobacter		0.66	–	–

Phylum/class	Genus	Gene	Normalized counts		
			SW	SI	SIO
	Sulfitobacter		–	0.55	–
Actinobacteria	*Nakamurella*		0.66	–	–
Chloroflexi	*Bellilinea*		0.66	–	–
Cyanobacteria	*Synechococcus*		2.65	1.10	–
Firmicutes	*Paenibacillus*		–	0.55	–
Alphaproteobacteria	*Ca. Pelagibacter*	*GSTK1*	1.32	1.10	2
	Gemmobacter		–	0.55	–
	Planktomarina		1.32	0.55	1
	Pseudochrobactrum		0.66	–	–
	Rhodoligotrophos		–	0.55	–
	Sphingobium		0.66	–	–
Betaproteobacteria	*Cupriavidus*		–	0.55	1
Gammaproteobacteria	*Bermanella*		–	–	1
	Ca. Thioglobus		0.66	0.55	1
Firmicutes	*Halocella*		–	0.55	–
Alphaproteobacteria	*Albidovulum*	*HPGDS*	–	0.55	–
	Altererythrobacter		–	0.55	–
	Ca. Pelagibacter		–	–	1
	Epibacterium		–	0.55	–
	Haematobacter				
	Mesorhizobium		0.66	–	–
	Planktomarina		3.31	2.76	2
	Planktotalea		–	–	1
	Tardiphaga		–	–	1
Betaproteobacteria	*Burkholderia*		–	0.55	–
Gammaproteobacteria	*Bermanella*		–	0.55	1
	Ca. Thioglobus		1.32	1.66	1
	Catenovulum		–	–	2
	Colwellia		–	–	2
	Marinomonas		–	0.55	–
	Methylophaga		–	0.55	–
	Oceanicoccus		0.66	–	1
	Parahaliea		–	0.55	–
	Thalassotalea		–	–	1
Alphaproteobacteria	*Antarctobacter*	*nahC*	–	0.55	–
	Ascidiaceihabitans		–	0.55	1
	Cohaesibacter		–	0.55	–
	Epibacterium		0.66	–	–
	Oceanicola		–	0.55	–
	Pannonibacter		0.66	–	–
	Planktomarina		0.66	–	–
	Planktotalea		1.99	0.55	1
	Pseudopelagicola		–	1.10	–
	Rugeria		–	0.55	–
Gammaproteobacteria	*Aliiglaciecola*		–	–	1
	Colwellia		–	0.55	–

Phylum/class	Genus	Gene	Normalized counts		
			SW	SI	SIO
Alphaproteobacteria	*Altererythrobacter*	*nidAB*	0.66	–	–
	Bradyrhizobium		–	0.55	–
	Brevundimonas		0.66	–	–
	Ca. Pelagibacter		2.65	2.76	5
	Ca. Puniceispirillum		2.65	1.10	1
	Cognatiyoonia		–	0.55	–
	Croceicoccus		–	0.55	–
	Enhydrobacter		0.66	–	–
	Epibacterium		–	0.55	–
	Hwanghaeella		–	0.55	–
	Methylobacterium		–	0.55	–
	Monaibacterium		–	0.55	–
	Pelagicola		0.66	–	–
	Planktomarina		3.97	1.10	2
	Pseudaestuariivita		–	0.55	–
	Pseudophaeobacter		–	0.55	1
	Roseovarius		0.66	1.10	–
	Ruegeria		0.66	–	–
	Sphingobium		0.66	0.55	–
	Sphingomonas		0.66	–	–
Gammaproteobacteria	*Alcanivorax*		–	0.55	–
	Ca. Thioglobus		5.30	6.63	3
	Cobetia		0.66	–	–
	Rudaea		0.66	–	–
	Woeseia		–	0.55	–
Actinobacteria	*Amycolatopsis*		0.66	–	–
	Streptomyces		1.32	–	–
Bacteroidetes	*Mangrovimonas*		0.66	–	–
Cyanobacteria	*Synechococcus*		0.66	–	–
Firmicutes	*Bacillus*		–	1.10	1

Table S13. Normalized counts (RPKG) of top100 prokaryotic genera associated with the genes or gene clusters from the gene group related to the degradation of various hydrocarbons. The associations based on the affiliation of the contigs containing sequences of respective genes in seawater (SW), sea ice (SI), and sea ice encapsulating crude oil (SIO) metagenomes. Archaea are given on green background.

Phylum/class	Genus	Gene	Normalized counts		
			SW	SI	SIO
Alphaproteobacteria	*Bradyrhizobium*	*chnB*	0.66	1.10	–
	Ca. Pelagibacter		7.95	9.39	12
	Ca. Thioglobus		1.32	1.10	–
	Hyphomonas		0.66	0.55	–
	Planktomarina		1.32	0.55	2
	Planktotalea		–	–	1
	Rhodobacter		–	0.55	1
	Sphingomonas		0.66	1.10	1
Betaproteobacteria	*Chromobacterium*		–	–	1
Deltaproteobacteria	*Sandaracinus*		–	–	1
Gammaproteobacteria	*Bermanella*		–	0.55	3
	Colwellia		–	0	1
	Glaciecola		–	0.55	2
	Photobacterium		–	–	1
	Pseudomonas		–	1.10	–
	Thalassotalea		–	–	1
	Umboniibacter		0.66	0.55	–
Actinobacteria	*Actinoplanes*		–	–	1
	Ilumatobacter		–	–	1
	Nonomuraea		0.66	1.10	–
	Pseudonocardia		–	1.10	–
	Saccharopolyspora		0.66	0.55	–
	Streptomonospora		0.66	0.55	–
	Streptomyces		0.66	0.55	–
Fibrobacteres	*Fibrobacter*		–	–	1
Firmicutes	*Bacillus*		–	1.10	2
Alphaproteobacteria	*Boseongicola*	*dbfA1A2*	0.66	0.55	–
	Ca. Pelagibacter		4.64	3.87	7
	Ca. Puniceispirillum		2.65	1.66	1
	Ca. Thioglobus		6.62	8.29	3
	Planktomarina		3.97	2.21	2
	Pseudophaeobacter		–	0.55	1
	Roseovarius		0.66	1.10	–
	Sphingobium		0.66	0.55	–
Actinobacteria	*Streptomyces*		1.32	–	–
Firmicutes	*Bacillus*		–	1.10	1
Alphaproteobacteria	*Reyranella*	*ligAB*	–	–	1
Betaproteobacteria	*Variovorax*		0.66	–	1
Gammaproteobacteria	*Arenicella*		–	–	1
	Parahaliea		–	–	1
Alphaproteobacteria	*Boseongicola*	*nagGH*	0.66	0.55	–

Phylum/class	Genus	Gene	Normalized counts		
			SW	SI	SIO
	Ca. Pelagibacter	*nagGH*	5.30	4.42	7
	Ca. Puniceispirillum		2.65	2.76	1
	Ca. Thioglobus		6.62	8.84	4
	Planktomarina		3.97	2.21	2
	Pseudooceanicola		0.66	0.55	1
	Pseudophaeobacter		–	0.55	1
	Roseovarius		0.66	1.10	–
	Sphingobium		0.66	0.55	–
Gammaproteobacteria	*Colwellia*		–	–	1
Actinobacteria	*Streptomyces*		1.32	–	–
Firmicutes	*Bacillus*		–	1.10	1
Alphaproteobacteria	*Ca. Pelagibacter*	*nahAabcd*	4.64	3.31	7
	Ca. Puniceispirillum		2.65	1.66	1
	Ca. Thioglobus		5.30	7.73	3
	Planktomarina		3.97	2.21	2
	Pseudophaeobacter		–	0.55	1
	Roseovarius		–	1.10	–
	Sphingobium		0.66	0.55	–
Actinobacteria	*Streptomyces*		1.32	–	–
Firmicutes	*Bacillus*		–	1.10	1
Alphaproteobacteria	*Agrobacterium*	*pcaGH*	0.66	0.55	–
	Ascidiaceihabitans		0.66	0.55	–
	Devosia		0.66	0.55	–
	Octadecabacter		1.32	0.55	2
	Planktomarina		1.32	0.55	2
	Sinorhizobium		0.66	0.55	–
	Sulfitobacter		–	0.55	1
	Tateyamaria		–	–	1
Alphaproteobacteria	*Ascidiaceihabitans*	*phdIJ*	0.66	1.10	–
	Ca. Pelagibacter		0.66	2.21	5
	Ca. Puniceispirillum		0.66	0.55	2
	Ca. Thioglobus		0.66	0.55	–
	Inquilinus		–	–	1
	Jannaschia		–	0.55	1
	Lentibacter		0.66	1.10	1
	Planktomarina		3.31	1.10	3
	Rhodobacter		0.66	1.10	–
Gammaproteobacteria	*Bermanella*		–	0.55	2
	Glaciecola		–	0.55	1
Actinobacteria	*Ca. Actinomarina*		–	–	1
	Microbacterium		–	–	1
Cyanobacteria	*Synechococcus*		2.65	1.10	–
Alphaproteobacteria	*Antarctobacter*	*yaiY*	–	–	1
	Ca. Pelagibacter		5.96	5.52	6
	Ca. Thioglobus		0.66	1.66	–
	Celeribacter		0.66	0.55	1

**Phylum/class**	**Genus**	**Gene**	**Normalized counts**		
			**SW**	**SI**	**SIO**
Alphaproteobacteria	*Epibacterium*	*yaiY*	0.66	–	1
	Oceaniovalibus		–	–	1
	Octadecabacter		0.66	0.55	1
	Planktomarina		1.32	1.66	1
	Ponticoccus		–	0.55	1
	Roseobacter		–	1.10	–
	Siccirubricoccus		–	0.55	1
	Sulfitobacter		–	0.55	1
Gammaproteobacteria	*Colwellia*		–	–	4
	Gallaecimonas		0.66	0.55	–
	Thalassotalea		0.66	0.55	1
	Umboniibacter		–	–	1
Thaumarchaeota	*Nitrosopumilus*		–	–	1

Table S14. The characteristics of all metagenome assembled genomes (MAGs) recovered from seawater (SW), sea ice (SI) and crude oil encapsulating sea ice (SIO) metagenomes. Red text indicates MAGs that fit within quality standards and were used in further analyses.

Metagenome	MAG	Completeness	Contamination
SW	SW 6B	100.00	749.47
	SW 10B	94.19	1.72
	SW 7B	83.11	1.46
	SW 15B	81.03	65.13
	SW 13B	77.85	3.14
	SW 5B	74.21	0.51
	SW 19B	71.43	3.73
	SW 8B	70.17	56.38
	SW 11B	69.89	23.57
	SW 9B	67.95	60.92
	SW 16B	66.79	0.68
	SW 1B	63.23	2.33
	SW 2B	46.55	1.72
	SW 3B	43.48	2.97
	SW 12B	34.99	0.00
	SW 17B	27.59	0.86
	SW 4B	18.81	4.39
	SW 14	16.14	0.00
	SW 18	0.00	0.00
	SW 1A	80.84	0.00
	SW 2A	17.76	0.93
SI	SI 19B	100.00	701.50
	SI 21B	94.45	2.03
	SI 18B	87.93	65.52
	SI 12B	76.76	1.97
	SI 20B	76.10	63.94
	SI 7B	75.74	36.05
	SI 15B	73.82	3.22
	SI 8B	73.72	12.53
	SI 24B	67.20	3.35
	SI 4B	53.06	1.68
	SI 23B	52.54	0.61
	SI 14B	51.01	11.72
	SI 22B	50.26	30.69
	SI 27B	50.00	1.72
	SI 28B	37.93	0.00
	SI 5B	35.90	0.50
	SI 3B	23.20	5.17
	SI 25B	19.30	1.75
	SI 17B	19.12	3.45
	SI 6B	17.41	0.00
	SI 1B	13.79	0.00
	SI 16B	12.50	0.00
	SI 2B	9.48	1.72

Metagenome	MAG	Completeness	Contamination
**SI**	SI 26B	8.77	0.00
	SI 13B	8.33	0.00
	SI 9B	5.17	0.00
	SI 11B	0.00	0.00
	SI 10B	0.00	0.00
	SI 1A	42.69	0.00
**SIO**	SIO 12B	98.46	1.01
	SIO 3B	95.30	204.11
	SIO 8B	81.03	3.45
	SIO 15B	77.90	81.3
	SIO 11B	68.39	19.86
	SIO 18B	57.41	16.95
	SIO 10B	51.37	20.19
	SIO 7B	50.00	5.17
	SIO 6B	24.91	3.64
	SIO 5B	22.97	7.18
	SIO 2B	22.56	4.66
	SIO 4B	17.53	0.49
	SIO 16B	10.34	0.00
	SIO 9B	8.33	4.17
	SIO 13B	8.33	0.00
	SIO 17B	0.86	0.00
	SIO 14B	0.00	0.00
	SIO 1B	0.00	0.00

Table S15. Taxonomy of good and high quality metagenome-assembled genomes assembled from metagenomes of seawater (SW), sea ice (SI), and crude oil encapsulating sea ice (SIO). Taxonomic level: k – kingdom; p – phylum; c – class; o – order; f – family; g – genus; s – species. B (bacteria) or A (archaea) in MAGs name indicate kingdom. Cut off value for ANIb was 95% and 90% for Kaiju. *More than 60% of contigs in MAG indicated to same genus or species.

Metagenome	MAG	Classification: CheckM	Classification: Kaiju (%)	Classification: JSpeciesWS (ANIb)
**SW**	SW 1B	(k) Bacteria	(p) Bacteroidetes (98.70)	–
	SW 5B	(o) Actinomycetales	(p) Actinobacteria (95.61)	–
	SW 7B	(f) Rhodobacteraceae	(f) Rhodobacteraceae (96.42)	(s) Rhodobacteraceae bacterium SB2 (99.03)
	SW 10B	(k) Bacteria	(p) Planctomycetes (96.17)	–
	SW 13B	(f) Rhodobacteraceae	(s) Planktomarina temperata (95.85)	(s) Planktomarina temperata RCA23 (98.43)
	SW 16B	(k) Bacteria	(k) Bacteria (100.00)	–
	SW 19B	(c) Gammaproteobacteria	(f) Halieaceae (91.67)	–
	SW 1A	(p) Euryarchaeota	(s) Candidatus Poseidoniales archaeon (90.41)	–
**SI**	SI 4B	(p) Proteobacteria	(g) Candidatus Thioglobus (90.15)	–
	SI 12B	(f) Rhodobacteraceae	(s) Planktomarina temperate (98.65)	(s) Planktomarina temperata RCA23 (98.29)
	SI 15B	(f) Rhodobacteraceae	(f) Rhodobacteraceae (98.45)	(s) Rhodobacteraceae bacterium SB2 (98.92)
	SI 21B	(f) Rhodobacteraceae	(f) Rhodobacteraceae (95.55)	–
	SI 23B	(o) Actinomycetales	(p) Actinobacteria (98.50)	–
	SI 24B	(p) Proteobacteria	(p) Proteobacteria (98.98) ***(s)Betaproteobacteria bacterium TMED22 (85.76)**	–
	SI 27B	(k) Bacteria	(c) Alphaproteobacteria (95.08)	–
**SIO**	SIO 7B	(k) Bacteria	(c) Gammaproteobacteria (96.89) ***(g) Bermanella (61.41)**	–
	SIO 8B	(k) Bacteria	(o) Alteromonadales (90.91) ***(g) Glaciecola (75.08)**	–
	SIO 12B	(f) Rhodobacteraceae	(f) Rhodobacteraceae (97.67)	–

Supplementary figures

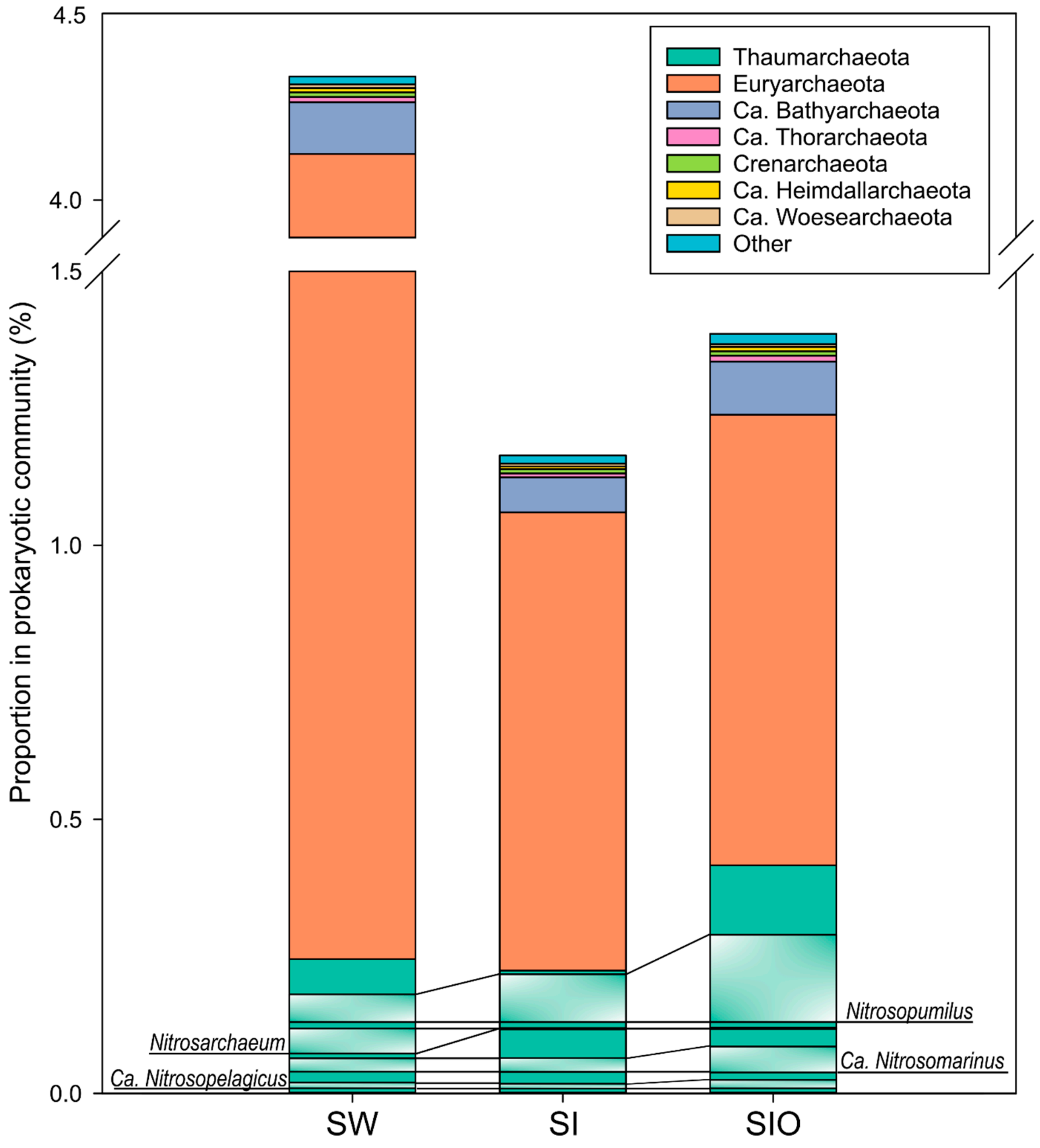

Figure S1. The proportions of archaeal phyla and most dominant archaeal genera in the prokaryotic community of seawater (SW), sea ice (SI), and crude oil encapsulating sea ice (SIO).
